# New Psychometric Evidence of a Bifactor Structure of the Emotional Regulation Questionnaire (ERQ) in Ecuadorian College Students

**DOI:** 10.11621/pir.2022.0108

**Published:** 2022-03-30

**Authors:** Rodrigo Moreta-Herrera, Mónica Perdomo-Pérez, Diego Vaca-Quintana, Hernán Sánchez-Vélez, Pamela Camacho-Bonilla, Fabricio Vásquez de la Bandera, Sergio Dominguez-Lara, Tomás Caycho-Rodríguez

**Affiliations:** a Pontificia Universidad Católica del Ecuador, Ambato, Ecuador; b Universitat de Girona, Girona, Spain; c Universidad de Ibagué, Ibagué, Colombia; d Universidad Tecnológica Indoamérica, Ambato, Ecuador; e Ministerio de Salud Pública del Ecuador, Quito, Ecuador; f Universidad Técnica de Ambato, Ambato, Ecuador; g Universidad de San Martín de Porres, Lima, Perú; h Universidad Privada del Norte, Lima, Perú

**Keywords:** Confirmatory Factor Analysis, emotion regulation, reliability, students, validity

## Abstract

**Background:**

Emotion Regulation comprises a set of strategies (cognitive, emotional, and physiological) that allow individuals faced with internal or external stimuli to manage their emotional response, to adapt to the environment, and to achieve goals. The Emotion Regulation Questionnaire (ERQ) is used to assess Emotion Regulation. It has been translated into several languages (including Spanish) and has been adapted around the world, but its psychometric properties have not been tested in Ecuador.

**Objective:**

To confirm the bifactor structure of the Emotion Regulation Questionnaire and its reliability in a sample of Ecuadorian college students.

**Design:**

A quantitative and instrumental study using Confirmatory Factor Analysis with Robust Maximum Likelihood estimation. The sample consisted of 400 participants (62.5% women), aged 18 to 25 (*M* = 21.1; *SD* = 1.95) from two universities in Ecuador and seven different undergraduate courses.

**Results:**

The bifactor model of the test is confirmed with an adequate adjustment ꭓ2 = 35.99; *p* > .001; ꭓ2/df = 1.43; CFI = .98; TLI = .96; SRMR = .034; and RMSE A = .033 CI_95%_: [.033–.052]; ω_H_ = .70; ω_Hs1_ = .23; ω_Hs2_ = .35. Reliability is high with ω = .86 CI_95%_: [.81–.88].

**Conclusion:**

The bifactor model of the ERQ is an adequate and reliable test to assess Emotion Regulation among Ecuadorian college students.

## Introduction

Emotion Regulation (ER) comprises a set of strategies (cognitive, emotional, and physiological) that allow individuals faced with internal or external stimuli to manage their emotional response, to adapt to the environment, and to achieve goals (Gross, 1999; Gross & John, 2003). Research in ER has grown exponentially due to the important role it plays in social adaptation and the development of certain psychopathologies (Aldao et al., 2010; Zumba-Tello & Moreta-Herrera, 2022), but also in the integral development of a person (Momeñe et al., 2017).

The Emotion Regulation Questionnaire (ERQ) (Gross & John, 2003) is used to evaluate ER; it is composed of 10 items and assesses two independent regulation strategies:

1. Cognitive Reappraisal (CR), an anticipatory strategy that allows reinterpretation and evaluation of context before the emotional response to modulate behavior when faced with triggering stimuli. Cognitive reappraisal is measured by six questions, such as: “When I want to feel a more positive emotion (such as joy or amusement), I change what I’m thinking about” or “When I want to feel a less negative emotion (such as sadness or anger), I change what I’m thinking about”.
2. Emotion Suppression (ES), which allows the modulation of emotions while the individual experiences them, is measured by four questions, such as: “I keep my emotions to myself ” or “When I am feeling positive emotions, I am careful not to express them” (Aldao et al., 2010; Balzarotti et al., 2010; Gross, 2007). The ERQ shows a Likert-type structure with seven response options, where 1 corresponds to “strongly disagree” and 7 corresponds to “strongly agree”.

The ERQ has been translated into several languages and validated around the world. Evidence of a two-factor orthogonal model (CR and ES without correlation) was presented in Italy (Balzarotti et al., 2010); Germany (Abler & Kessler, 2009); Spain (Cabello et al., 2013; Rodríguez-Carvajal et al., 2006); Portugal (Teixeira et al., 2015); Australia and the United Kingdom (Spaapen et al., 2014), and the USA (Preece et al., 2021); while studies showed evidence of a two-factor oblique model (CR and ES correlated) in Sweden (Enebrink et al., 2013); Peru (Gargurevich & Matos, 2010); Ecuador (Moreta-Herrera et al., 2018; Moreta-Herrera et al., 2021a), and Australia (Preece et al., 2020). Both two-factor adjustment models (orthogonal and oblique), present adequate internal consistency reliability as well as convergent validity when compared with other tests (health, well-being, emotional intelligence, among others). In the case of Ecuador, no studies on the factorial structure of the ERQ have been found in the scientific literature, which raises the importance of the present research.

### Methodological Implications of the Validation of Tests

Having tests translated, adjusted, and adapted to the context in which the ERQ or any other test is applied is one of the challenges of evidence-based instrumental research. Contemporary empirical research has focused more on social and psychic phenomena than on the development and validation of assessment tools. The use of assessment tools without proper instrumental validation can compromise results from the beginning, due to the absence of calibration (Moreta-Herrera et al., 2019), which leads to measurement errors and biases (Elosua, 2003). This can also cause errors in decision making, testing hypotheses, and diagnosis (Rönkkö et al., 2015).

Many researchers do not report the proper nature of the test items (commonly a Likert-type scale), which is problematic, since depending on the number of options, they may assume an ordinal (five options or less) or continuous (more than five options) nature. This is relevant because multivariate normality is usually less likely in the former. In addition, the absence of multivariate normality is very common in social science research (Jin & Cao, 2018; Li, 2016). This results in the incorrect use of statistical tests during the validation processes, which do not correspond to the nature of the items or the assumption of multivariate normal distribution (Sullivan & Artino, 2013). These errors are observed in different statistical validation and reliability processes such as Exploratory Factor Analysis (EFA) with Principal Components Analysis (PCA), Confirmatory Factor Analysis (CFA) with Maximum Likelihood (ML), internal consistency reliability with Cronbach’s alpha (α), among others.

### Considerations in Confirmatory Factor Analysis and Reliability

CFA is a statistical method widely used as evidence for the construct validity of a measure (Ferrando & Anguiano-Carrasco, 2010). It requires a considerable sample size (Brown, 2015), the confirmation of multivariate normality (Cain et al., 2017), and the nature of the variables (categorical, ordinal, or interval) (Hair et al., 2004). The treatment of data and the decision whether to employ normal or robust estimators will depend on whether these criteria are met.

CFA is generally calculated with the Maximum Likelihood Estimation method (ML) (Li, 2016), which assumes that the observed indicators (items) follow a continuous and multivariate normal distribution (Myung, 2003). In the case of psychological tests, this is not the most suitable method, as items usually have an ordinal nature (Gitta & Bengt, 2009) and continuous multivariate normal distribution is unlikely (Holtmann et al., 2016). Therefore, CFA requires estimators appropriate to these characteristics such as the Diagonally Weighted Least Squares (DWLS) method or robust estimations such as Robust Maximum Likelihood (MLR) or Weighted Least Squares with Adjusted Mean and Variance (WLSMV) (Jin & Cao, 2018). These methods, especially MLR, are recommended, as they reduce biases compared to ML. This helps to obtain stronger evidence of validity, regardless of the number of categories of the item and without multivariate normal distribution as long as a large sample size is analyzed (*n* > 200) (Li, 2016).

Previous studies confirm an orthogonal two-factor model (Abler & Kessler, 2009; Balzarotti et al., 2010; Cabello et al., 2013; Preece et al., 2021; Rodríguez-Carvajal et al., 2006; Spaapen et al., 2014; Teixeira et al., 2015) of the ERQ (Gross & Jhon, 2003), although an alternative configuration of an oblique two-factor model is also proposed (Enebrink et al., 2013; Gargurevich & Matos, 2010; Moreta-Herrera et al., 2018; Preece et al., 2020). The different configurations of the models in these studies are probably due to particular characteristics of the reference samples, differences in language, and the estimators used in factor analysis (ML estimation is predominant in validation studies, which induces a greater measurement bias) (Caycho-Rodríguez et al., 2021; Jonason et al., 2020; Moreta-Herrera et al., 2021b).

Due to the presence of moderate factor correlations in preliminary studies, there is likely to be a third latent factor that groups all the items of the scale into a single factor; this would be explained through a bifactor model composed of a general factor (GF) and two specific factors (SF). This model best represents the multidimensionality of the construct and recognizes the uniqueness of the factors that compose it, but also the binding capacity of the items in a general factor (Stefansson et al., 2016), allowing a better interpretation of the factors as well as a global reading of the construct, so its use is becoming more common in validation studies. In the case of the ERQ, there is no preliminary evidence of a bifactor adjustment model.

Something similar occurs when determining the internal consistency reliability of the ERQ through Cronbach’s alpha coefficient (α) (Sijtsma, 2009), a test that requires a significant number of cases for its analysis, as well as a continuous multivariate normal distribution. However, evidence suggests that using Cronbach’s alpha is not ideal for this purpose (Trizano-Hermosilla & Alvarado, 2016), due to the ordinal nature of the items; Cronbach’s alpha does not consider this aspect, and its use is recommended only when the measurement scale has six or more options and the normal distribution assumption is met (Elosua Oliden & Zumbo, 2008). As a result, researchers underestimate or overestimate the true reliability of the measure; therefore, its use is not recommended (Ventura-León & Caycho-Rodríguez, 2017). Given this situation, it is methodologically correct to use reliability estimators according to the nature of the items, such as the omega coefficient (McDonald, 1999), which shows less bias in the assessment of reliability (Dunn et al., 2014), or the ordinal coefficient alpha (Elosua Oliden & Zumbo, 2008).

Given these antecedents, there are still doubts that still need to be clarified about the best factorial fit of the ERQ, as well as other psychometric properties such as reliability, for their correct use in social research and intervention, especially in the Latin American and Spanish-speaking population.

### Objectives and Hypotheses

**Figure 1.**
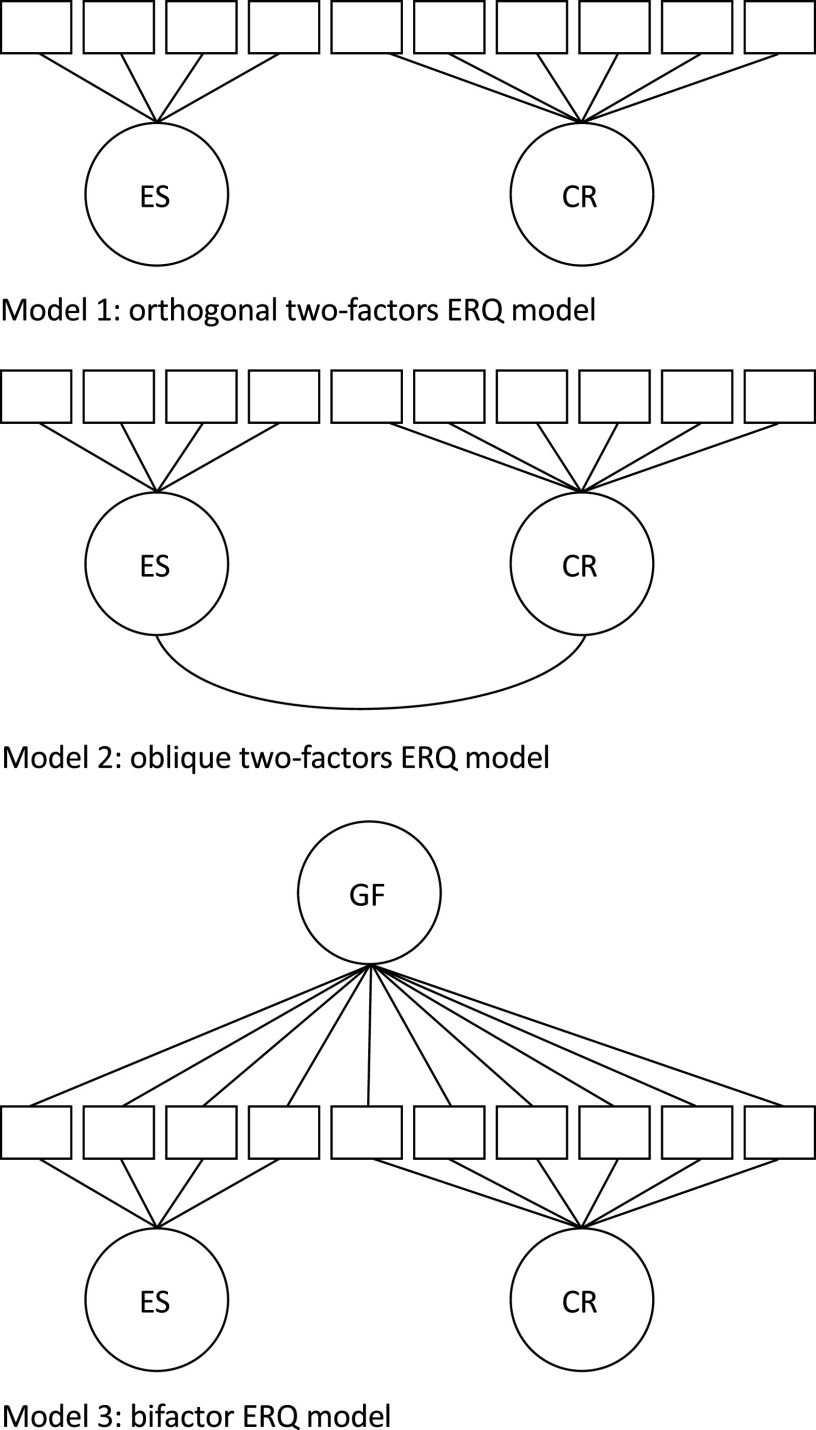
Different Models of the Emotion Regulation Questionnaire Evaluated in the Study

Based on the analysis contained in this text, the objectives of this study are a) to confirm the bifactor structure of the Emotion Regulation Questionnaire, comparing an orthogonal and an oblique two-factor model as well as a bifactor model with a general factor (see Figure 1) in a sample of Ecuadorian college students. It is hypothesized that the bifactor model is the model with the best fit; b) to estimate the internal consistency reliability of the ERQ model with the best fit. It is hypothesized that the ERQ has an optimal and adequate adjustment for Ecuadorian college students.

## Method

This study applied a quantitative and instrumental descriptive design (Ato et al., 2013) to confirm the model of two correlated factors of the ERQ in a sample of Ecuadorian college students through appropriate statistical tests for ordinal variables.

### Participants

Our sample included 400 college students, aged 18 to 25 years (*M* = 21.1 years; *SD* = 1.95), where 62.5% are women and 37.5% are men. In terms of ethnicity, most identified as mestizos (97.8%), while a few identified as white or indigenous (2.3%). In addition, 86% are located in urban areas and 14% in rural areas. Participants are students from two universities in Ambato, Ecuador; one public (62.5%) and one cofinanced (37.5%), and from seven different undergraduate courses. Finally, 36.8% of the sample receive financial aid, and 3.1% present academic risk due to poor performance.

Participants were selected through a non-probabilistic convenience sampling with the following inclusion criteria: a) voluntary participation through a signed consent letter; b) enrollment and regular class attendance; and c) adequate mental health to carry out the psychological evaluation process.

### Procedure

After permission was given by the authorities of the participating universities, the psychological evaluation began. All students interested in the research project were summoned to receive information about the objectives of the study and the activities they would perform. Before the general evaluation, a pilot test was carried out with 30 participants to learn details about the evaluation time and language adaptations that could be necessary for the items of the test.

Once in the global evaluation, participants signed a letter of consent before beginning the psychological assessment. After the evaluation, data was refined and digitized for subsequent statistical analysis and hypothesis verification. With the results achieved, the written report was prepared and approved.

### Instrument

*Emotion Regulation Questionnaire* (ERQ; Gross & John, 2003) in its Spanish version (Rodríguez-Carvajal et al., 2006) and adapted to Ecuadorian college students (More-ta-Herrera et al., 2018). It has 10 items measured on a five-point Likert scale, ranging from strongly disagree (1) to strongly agree (7), in which Cognitive Reappraisal and Emotion Suppression strategies are measured.

### Data Analysis

Data analysis was divided into three blocks. The first block corresponded to preliminary analysis, to learn the behavior of the variables using measures of central tendency, dispersion, and distribution. The univariate normality assumption was verified due to the values of skewness and kurtosis being within the parameter ±1.5 (Ferrando & Anguiano-Carrasco, 2010). Finally, the assumption of multivariate normality was checked through the Mardia test, where skewness and kurtosis were found to be not significant (*p* > .05) (Cain et al., 2017; Mardia, 1970).

The second block corresponded to the CFA with the RML estimator, which is reported as the most appropriate estimator considering the continuous nature of the variables and the absence of multivariate normality (Holtmann et al., 2016; Jin & Cao, 2018). Three models have been tested: a) an oblique two-factor model; b) an orthogonal two-factor model; and c) a bifactor model with two specific factors (SF) and a general factor (GF). The analysis verified that standardized factor loadings were λ > 0.5, which positively contributes to the explained variance (Hair et al., 2004). Different adjustment levels were also analyzed: a) absolute fit indices through the Chi-squared test (*X*^2^), normed Chi-square (*X*^2^/*df*), and the Standardized Root Mean Square Residual (SRMR); b) relative fit indices such as the Comparative Fit Index (CFI) and the Tucker-Lewis Index (TLI); and c) a non-centrality-based index through the Mean Square Error of Approximation (RMSE A). A model has an adequate adjustment when *χ*^2^ is not significant (*p* > .05) or *χ* ^2^/*df* is less than 4, CFI and TLI are greater than 0.9, and SRMR together with RMSE A are less than 0.08 (Brown, 2015; Byrne, 2008; Ferrando & Anguiano-Carrasco, 2010; Mueller & Hancock, 2018; Wolf et al., 2013). For the bifactor model, the Hierarchical Omega adjustments for the general factor (ω_H_), the specific factors (ω_Hs_), and the Common Explained Variance (ECV) were also tested. The bifactor model presented an adequate adjustment with ω_H_ > = .70, ECV > = .70, and the ω_Hs_ > = .30 (Reise et al., 2013; Rodríguez-Lara & Rodríguez, 2017; Rodriguez et al., 2016).

The third block included analysis of internal consistency of the ERQ using the omega coefficient (ω, McDonald, 1999; Ventura-León & Caycho-Rodríguez, 2017), together with the confidence intervals that ensure a better estimate of internal consistency (Domínguez-Lara & Merino-Soto, 2015). All data analyses were performed using R software (R Core Team, 2019), an open-access program.

## Results

### Preliminary Analysis

*Table 1* shows that the item scores are generally concentrated in the middle of the response scale, displaying a moderate distribution. Univariate normality analysis shows that this assumption is fulfilled based on the fact that both skewness and kurtosis scores are within the normal range (±1.5); while the assumption of multivariate normality is not met since the Mardia test shows significance for both skewness and kurtosis.

**Table 1:** Preliminary Analysis of the ERQ Items

Item		*M*	*SD*	Skew	Kurt
Cuando quiero incrementar misemociones positivas (p.ej. alegría, diversión), cambio el tema sobre el que estoy pensando.	When I want to feel more positive emotion (such as joy or amusement), I change what I’m thinking about	4.84	1.65	–0.79	–0.14
Guardo mis emociones para mí mismo	I keep my emotions to myself	4.71	1.69	–0.61	–0.46
Cuando quiero reducir mis emociones negativas (p.ej. tristeza, enfado), cambio el tema sobre el que estoy pensando	When I want to feel less negative emotion (such as sadness or anger), I change what I’m thinkingabout	5.05	1.56	–0.74	–0.21
Cuando estoy sintiendo emociones positivas, tengo cuidado de no expresarlas	When I am feeling positive emotions, I am careful not to express them	3.58	1.71	0.12	–1.03
Cuando me enfrento a una situ- ación estresante, intento pensar en ella de un modo que me ayude a mantener la calma	When I’m faced with a stressful situation, I make myself think about it in a way that helps me stay calm	4.85	1.67	–0.70	–0.40
Controlo mis emociones no expresándolas	I control my emotions by not expressing them	4.04	1.71	–0.12	–0.85
Cuando quiero incrementar mis emociones positivas, cambio mi manera de pensar sobre la situación	When I want to feel more positive emotion, I change the way I’m thinking about the situation	4.68	1.60	–0.65	–0.21
Controlo mis emociones cambi- ando mi forma de pensar sobre la situación en la que me encuentro	I control my emotions by changing the way I think about the situation I’m in	4.79	1.45	–0.52	–0.09
Cuando estoy sintiendo emocio- nes negativas, me aseguro de no expresarlas	When I am feeling negative emotions, I make sure not to express them	4.39	1.62	–0.30	–0.60
Cuando quiero reducir mis emociones negativas, cambio mi manera de pensar sobre la situación	When I want to feel less negative emotion, I change the way I’m thinking about the situation	4.86	1.56	–0.67	–0.13
		**Mardia**		951.8***	29.2*

Note. * p < .05; *** p < .001; M: sample mean; SD: standard deviation; Skew: skewness; Kurt: kurtosis

### Confirmatory Factor Analysis

*Table 2* shows the results of the fit indices of the three models of the ERQ evaluated in this study. The first model is the original one proposed by Gross & Jhon (2003); the second one is the oblique two-factor model; and the third corresponds to the bifactor model. Applying the MLR estimator, the oblique two-factor model (with a moderate latent correlation of ρ = .56) and the bifactor model of the ERQ presents an adequate adjustment as shown by absolute fit indices (*χ* ^2^, *χ* ^2^/*df*, SRMR), relative fit indices (CFI, TLI), and non-centrality-based index (RMSE A). The fit values for the bifactor model are better than those of the oblique two-factor model. The ANOVA function for SEM carried out by the Satorra-Bentler scaled Chi-square difference test (Satorra & Bentler, 2001) identifies the differences of adjustment of the Chi-squared and presents significant differences (*p* < .05) between the models, with ꭓ^2^(bifactor – oblique two-factor) = 59.26; *df* (bifactor – oblique two-factor) = 9; *p* <.001, so the bifactor model is a better fit than the oblique two-factor model.

**Table 2:** Confirmatory Factor Analysis of the ERQ with MLR Estimation

Models	*ꭓ* ^2^	*df*	***ꭓ*^2^/** *df*	CFI	TLI	SRMR	RMSEA
Orthogonal two factors	154.12***	35	4.40	.83	.79	.17	.09 [.08–.11]
Oblique two factors	99.45***	34	2.93	.91	.90	.06	.07 [.06–.08]
Bifactor	35.99	25	1.43	.98	.96	.03	.03 [.01–.05]

*Note. ꭓ^2^: Chi-squared test; df = degrees of freedom; χ^2^/df: normed Chi-square; CFI: Comparative Fit Index; TLI: Tucker-Lewis Index; SRMR: Standardized Mean Square Residue; RMSEA: Mean Square Error of Approximation*

**Figure 2.**
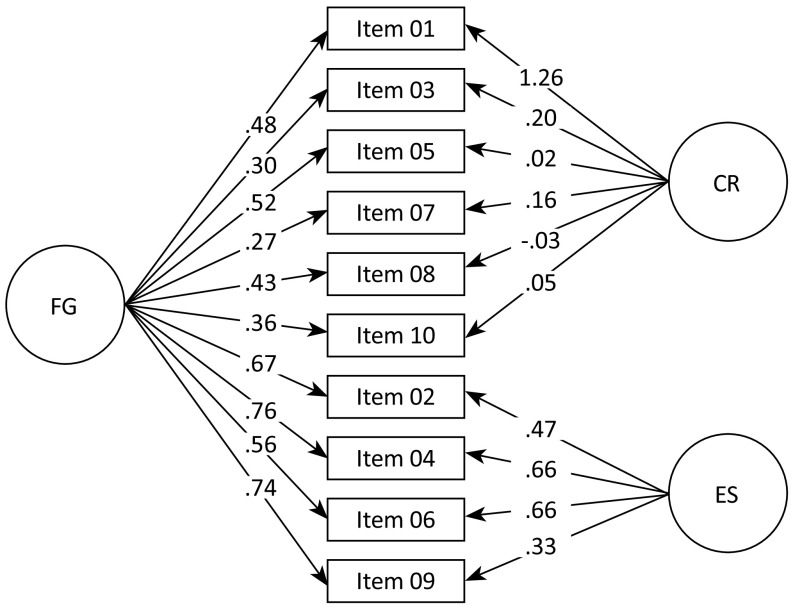
Bifactor Model of the Emotion Regulation Questionnaire

Regarding the CFA of the ERQ, factor loadings of the bifactor model were tested. Figure 2 shows that the behavior of standardized factor loadings (λ) through the general factor is more consistent than through the specific factors of the ERQ; therefore, the general factor presents a better explained variance than the specific factors. This is confirmed with better adjustment of the ω_H_ and moderate adjustment of the ECV and PUC for the general factor when compared to the specific factors.

ωH = .70; ωHs1 = .23; ωHs2 = .35; ECV = .50; PUC = .53

*Note: Circles represent the latent variables that comprise errors and factors (Cognitive Reappraisal and Emotion Suppression), while rectangles represent the observed variables that are the test items*.

### Reliability Analysis

*Table 3* presents the omega coefficient (ω) values with their respective confidence interval of each of the ERQ factors, which report an acceptable degree of internal consistency; this is evidence that the ERQ is a reliable instrument for Ecuadorian college students. Furthermore, the intercorrelations of the ERQ factors with their overall score show that the factors have moderate and high levels of correlation, so it is estimated that they contribute significantly to the model.

**Table 3:** Analysis of Reliability and Intercorrelations of the ERQ

Factor	ω CI 95%	CR	ES	ERQ
Cognitive Reappraisal	.85 [.83–.87]	1	.303**	.713**
Emotion Suppression	.75 [.71–.79]		1	.624**
Global	.86 [.84–.88]			1

Note. ^**^ p < .01; ω: McDonald´s omega coefficient; CR: Cognitive Reappraisal; ES: Emotion Suppression; ERQ: Emotion Regulation Questionnaire

## Discussion

The objectives of this study were to identify the best adjust model of the ERQ, as well as its reliability in a sample of Ecuadorian college students. Regarding the CFA procedure, given the absence of multivariate normality and the continuous distribution of the observed variables (see Table 1), the use of a robust estimator was necessary (Gitta & Bengt, 2009; Holtmann et al., 2016). Robust Maximum Likelihood estimation (MLR) was chosen, since this method presents the best results in the cases indicated for its use (Li, 2016). In addition, the use of MLR is justified not only in the preliminary criteria to the CFA, but also due to its recent use in similar validation processes of the ERQ (Preece et al., 2020).

CFA with MLR estimation found that the oblique two-factor and the bifactor models are optimum and consistent. Absolute Fit Indices (*χ*^2^, *χ*^2^/*df* and SRMR), Relative Fit Indices (CFI, TLI), and the non-centrality-based index (RMSE A) (Brown, 2015; Byrne, 2008; Ferrando & Anguiano-Carrasco, 2010; Mueller & Hancock, 2018; Wolf et al., 2013) reflect adequate values. This confirms the good fit of the ERQ for Ecuadorian college students. The results presented in this study are consistent with those presented previously (Enebrink et al., 2013; Gargurevich & Matos, 2010; More-ta-Herrera et al., 2018; Preece et al., 2020), and differ from the orthogonal two-factor model proposed by Gross & Jhon (2003) and from other similar validation studies (Abler & Kessler, 2009; Balzarotti et al., 2010; Cabello et al., 2013; Preece et al., 2021; Rodríguez-Carvajal et al., 2006; Spaapen et al., 2014; Teixeira et al., 2015), since the orthogonal two-factor model did not present a relevant fit.

Likewise, there is a latent interfactorial correlation in the oblique model (ρ), which allows exploring a new multidimensional model through a bifactor model, which encompasses all its items in a general factor, while respecting the uniqueness of the specific factors (Stefansson et al., 2016). This model has better factorial configuration settings (Reise et al., 2013; Rodriguez et al., 2016; Rodríguez-Lara & Rodríguez, 2017) and differs significantly from the previous model (*X*^2^(bifactor – oblique two-factors) = 59.26; *df*(bifactor – oblique two-factors) = 9; *p* < .001); consequently, its use is recommended. This is relevant in psychometric research because it proposes a multidimensional model of which there are no previous reports. This will allow in the future new processes of normalization of the scores considering the global result of the test, which was previously inadequate, and reveals an unexplored composition of this assessment tool that maximizes the interpretation of the construct Emotion Regulation. However, since these findings do not yet have supporting evidence, they should be viewed with caution pending future confirmatory studies.

Regarding reliability, it was found that both McDonald’s coefficient scores and their confidence intervals (CI) are within accepted parameters (Domínguez-Lara & Merino-Soto, 2015; Ventura-León & Caycho-Rodríguez, 2017), with both of the internal components (Cognitive Reappraisal and Emotion Suppression) and with the global assessment. In the context of Ecuador, these results (CFA and reliability) share similar conclusions to those of previous research of Moreta-Herrera et al. (2018) with psychology students. However, due to the modification of the methodology, it is necessary to be cautious with future comparisons because there are no similar studies that serve as a reference.

## Conclusion

Both CFA with RML estimation and reliability through McDonald’s coefficient (1999) of the ERQ bifactor model show adequate validation results. Thus, there is sufficient evidence of validity (Elosua, 2003) for the use of the ERQ in research and diagnosis in samples of Ecuadorian college students. Given the methodological variants used at the time of this analysis, new confirmatory studies are required to verify the factorial structure of the ERQ in other contexts.

Within the implications of the present study for instrumental research, the gate is open for the strengthening of this line of research in Ecuador and the region. An updated methodological framework is offered, and its use is recommended for validation processes of psychological tests. Three innovations are presented: a) CFA with a robust method (MLR); b) the omega coefficient (ω) for internal consistency with the confidence intervals; and c) a new factor configuration of the scale. The first two are recommended for an adequate analysis for continuous variables that do not present normal distribution, and the third one to improve the assessment of the real reliability of a test. Finally, the results obtained in the ERQ analysis allow us to confirm that it shows good validity in terms of factorial structure and high reliability.

## Limitations

One of the main limitations of this study is related to the lack of other validation processes such as convergent and discriminant validity, which were not carried out due to limitations inherent to the study, since no information was collected that would allow this process. For future research, it is recommended to take this aspect into account for more in-depth studies. This study only analyzes the factorial validity of the ERQ test, but not the measurement invariance for multigroup studies (culture, sex, age groups, and others). Therefore, this should be considered and confirmed in advance as a preliminary step for comparative studies. Finally, only students from two universities in Ecuador were considered; therefore, we recommend replicating this study with other types of populations such as adolescents, the general population, and others.
